# An enhanced recyclable 3D adsorbent for diverse bio-applications using biocompatible magnetic nanomulberry and cucurbituril composites

**DOI:** 10.1038/s41598-019-57336-4

**Published:** 2020-01-16

**Authors:** Yange Luan, Huifang Liu, Zhen Qiao, Bonhan Koo, Jaehyub Shin, Yoon Ok Jang, Jin-Seo Noh, Yong Shin

**Affiliations:** 1 Department of Convergence Medicine, Asan Medical Institute of Convergence Science and Technology (AMIST), University of Ulsan College of Medicine, Biomedical Engineering Research Center, Asan Institute of Life Sciences, Asan Medical Center, 88 Olympicro-43gil, Songpa-gu, Seoul, Republic of Korea; 2 Department of Nano-Physics, Gachon University, 1342 Seongnamdaero, Sujeong-gu, Gyeonggi-do 13120 Republic of Korea

**Keywords:** Biomedical materials, Pollution remediation

## Abstract

Herein, we describe the synthesis of highly water-dispersible and biocompatible 3D adsorbents via a rapid two-step strategy employing a mesoporous magnetic nanomulberry-shaped Fe_3_O_4_ (MNM) on diatomaceous earth (DE) and cucurbituril (CB; MNM-DE-CB). Coating of CB on the surface of MNM-DE via hydrogen bonds not only enhanced the dispersibility of CB, but also improved the stability of MNM-DE. The ability of the adsorbent to remove dyes from water was investigated as a function of metal ions, solution pH, temperature, and concentration to determine optimum reaction conditions. Unlike MNM-DE, MNM-DE-CB exhibited highly efficient, rapid dye removal and recyclability in aqueous solution, and low cytotoxicity toward cancer cells in drug delivery tests. MNM-DE-CB is a promising green adsorbent with potential for diverse applications including water remediation, interface catalysis, bio-sample preparation, and drug delivery.

## Introduction

Numerous nanotechnology-based techniques for the treatment of wastewater and drugs have so far only been investigated at the laboratory scale due to barriers including low efficiency, dissolubility, toxicity, and non-reusability^1–4^. Among emerging technologies, nano-sorbents and activators have received considerable attention^5,6^. Nanomaterials are highly valued as sorbents, and modification of their large surface area with specific reactive groups can increase their affinity toward particular target compounds^7,8^. Nanomaterial-based physical, chemical, and catalytic adsorbents for ecofriendly-efficient applications should be fabricated conveniently, collected easily after usage, and ideally recycled and reused^9,10^. Therefore, the development of functional natural resources provides a new challenge and an opportunity for industrial production.

Diatomaceous earth (DE) has an extraordinary 3D porous structure with micro- to nanoscale dimensions that has allowed it to be successfully employed in photonics, sensing, biosensing, filtration, adsorption, microfluidics, catalysis, drug delivery, and nanofabrication^11,12^. The high biocompatibility, reproducibility, and low production cost of DE makes it attractive for many applications, and its highly modifiable surface is a significant advantage for nano-sorbents. However, exploration of pure DE as a commercial sorbent has been limited by the loading capacity due to its size^13^. Furthermore, repeated usability (and preferably cyclic utilization) is essential for eco-friendly wastewater treatment systems^14,15^. Therefore, considerable research effort has been invested in modifying the DE structure into technologically more suitable functional materials. To expand the usefulness of DE and overcome its inherent limitations, two types of materials have emerged as promising candidates: super-molecular cucurbiturils (CBs) with high molecular absorption properties, and magnetic materials with potential for treating wastewater at a large scale within a short time due to facile collection with an external magnet^11,16–18^. Pumpkin-shaped CBs are important in host–guest chemistry and have been employed for molecular encapsulation, water treatment, surface adhesion, biomarker-targeted theranostics, and drug delivery^19–21^. Driven by a diverse range of inter- and intramolecular interactions, CBs are ideal hosts for charged amphiphilic guests due to ion-dipole stabilization and possible hydrogen bonding inside the CB cavity^20,22^. Although the great potential of CBs has been highlighted in many studies, three major performance limitations remain to be addressed: (1) unexplained poor solubility in aqueous solutions, (2) unsuccessful functional group modification, and (3) unclarified ion effects in CB applications^23–25^. In particular, poor solubility poses a serious obstacle for the development of CB-based applications, and much research effort has been expended to overcome these limitations by developing new water-soluble host/guest systems with a synergistic blend of supra-molecular assemblies and nanomaterials. Additionally, magnetic nanocrystals are of importance in various applications, as they can be used for both tracing and diverse valence configurations in colloid interface science and functional materials^2,8,11^. Mesoporous materials have attracted much attention for many applications such as catalysts, catalysis supports, drug carriers, electric materials, and adsorbents due to the narrow pore size distribution and high surface areas^26–29^. Hence, mesoporous based magnetite (Fe_3_O_4_) nanomaterials have been widely utilized in many fields including sensing agents, catalysis, electrical devices, energy storage devices, and biomedical drug loading and delivery^30–34^. However, Fe_3_O_4_ nanoparticles often suffer from poor stability and dispersity, which represents a barrier to advanced applications.

In the present work, we prepared a novel adsorbent consisting of mesoporous magnetic nanomulberry-shaped Fe_3_O_4_ (MNM) on the surface of DE and CB (MNM-DE-CB) via a facile two-step method. Subsequent characterization demonstrated the ability to synergize the stability and solubility of the adsorbent for pollutant removal and encapsulating molecules in aqueous solution. MNM-DE-CB can be easily collected and recycled due to its strong magnetic properties, reducing reagent costs. The structural characteristics of the MNM-DE-CB composite, adsorption capacity, and influence of metal ions and pH were evaluated. Finally, the 3D adsorbent was subjected to cytotoxicity testing using a cancer drug to demonstrate drug delivery applications.

## Results and Discussion

### Principle of MNM-DE-CB

**Figure 1:**
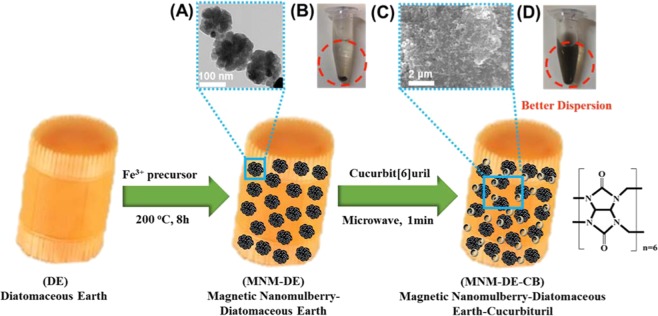
Schematic illustration of the preparation of magnetic nanomulberry-diatomaceous earth-cucurbituril (MNM-DE-CB). (**A**) Transmission electron microscopy (TEM) image of MNM. Scale bar = 100 nm. (**B**) MNM-DE in aqueous solution. (**C**) Scanning electron microscopy (SEM) image of MNM-DE-CB. Scale bar = 2 μm. (D) MNM-DE-CB in aqueous solution.

A novel two-step fabrication of MNM-DE-CB was developed (Fig. 1) to extend the usefulness of natural DE. First, DE with a uniform particle size distribution (10−25 μm) was thoroughly washed and combined with magnetic material using the traditional hydrothermal synthesis method^35,36^. The structure and high magnetization of the synthesized magnetic nanomulberry-shaped CB on DE (MNM-DE) were confirmed by TEM, SEM, and VSM (Fig. 1A and Fig. S1A,B). Although an increase in the surface area of MNM-DE composites was expected, no significant changes occurred due to the facile coagulation of MNM-DE composites in solution (Fig. 1B) possibly caused by interactions between magnetic forces on neighboring magnetized compounds. Further surface modification of MNM-DE was then implemented using super-molecular cucurbit[6]uril (CB[6]) to enhance the functionality of nanomaterial composites in aqueous solution. CB[6] solution was mixed with the prepared MNM-DE and boiled for 1 min using a microwave. After two-step fabrication, the structure of MNM-DE-CB was confirmed using SEM (Fig. 1C). MNM-DE-CB dispersed much better in aqueous solution (Fig. 1D) than MNM-DE (Fig. 1B). In addition, the novel porous structure of MNM-DE-CB gained magnetization and enhanced the surface area of the composites. To confirm the improvements for MNM-DE-CB, we deduced that CB[6] powder gradually dissolved due to interactions with hydrophilic groups on the surface of MNM when insoluble CB[6] encountered MNM-DE in solution^23,37^ We confirmed that active ions in the MNM-DE solution accelerated CB binding and/or hosting on the surface of MNM-DE using energy-dispersive X-ray spectroscopy (EDX; Fig. S1C,D). A delicate dynamic system between CB and MNM-DE was evident, which also verified the improved dissolvability and dispersibility of MNM-DE-CB composites in aqueous solution.

### Characterization and analysis of MNM-DE-CB

**Figure 2:**
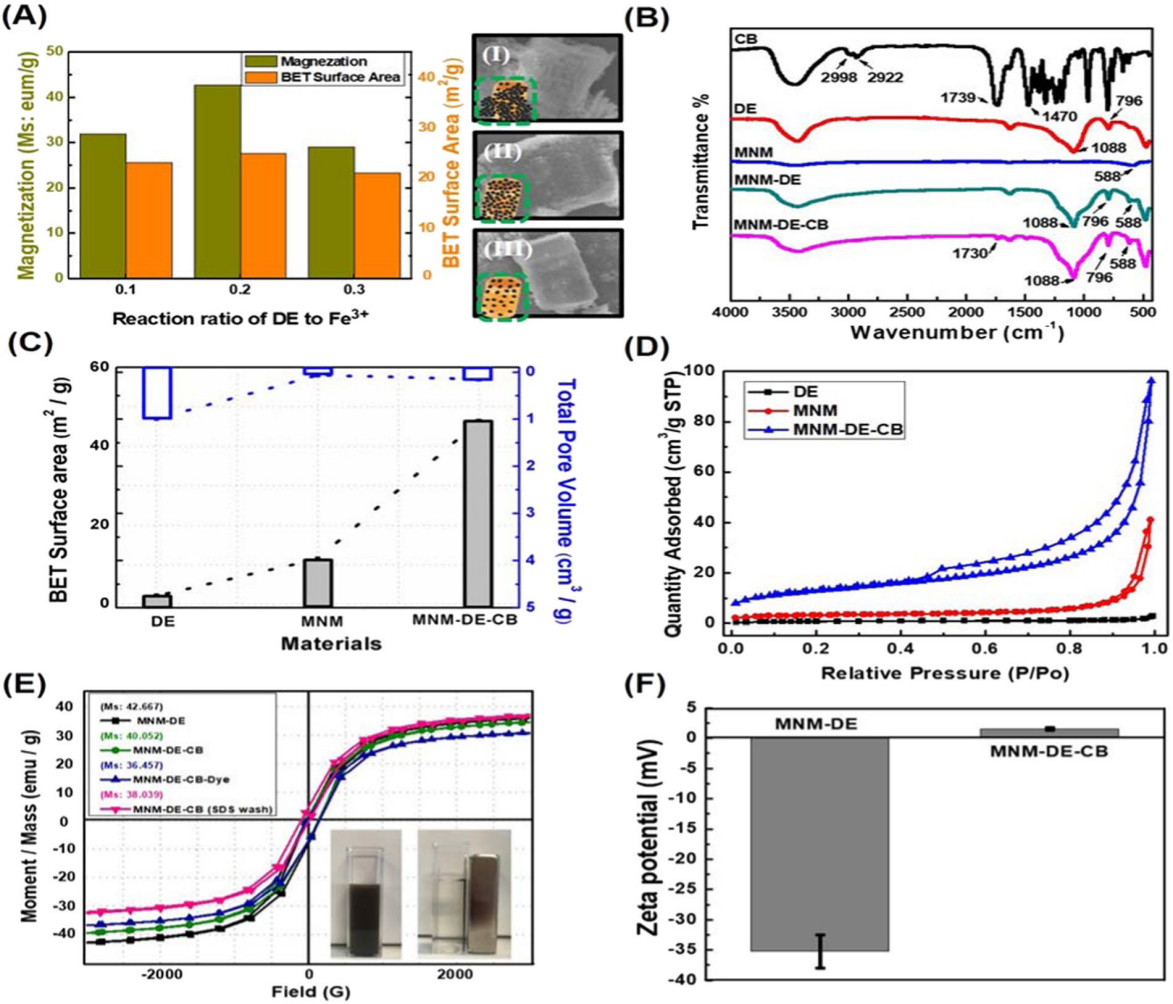
Characterization of MNM-DE-CB. (**A**) Magnetization, Brunaer-Emmett-Teller (BET) surface area, and SEM images of MNM-DE at three different reaction ratios of DE to Fe3 + : 0.1 (I), 0.2 (II), and 0.3 (III). Samples of 0.3 g, 0.2 g, and 0.1 g pure DE were added to a 20 mL reaction system to obtain the desired ratio. (**B**) Fourier-transform infrared (FTIR) spectroscopy analysis of CB (black line), DE (red line), MNM (blue line), MNM-DE (green line), and MNM-DE-CB (pink line). (**C**) BET surface area and total pore volume of pure DE, MNM, and MNM-DE-CB. (**D**) Nitrogen adsorption-desorption isotherm curve analysis of prepared DE (black line), MNM (red line), and MNM-DE-CB (blue line). (**E**) Magnetic hysteresis loops of MNM-DE, MNM-DE-CB, MNM-DE-CB-dye (MNM-DE-CB after absorbing dye), and MNM-DE-CB (MNM-DE-CB-dye after washing with SDS). The insets show the behavior of MNM-DE-CB under an external magnetic field at room temperature. (**F**) Zeta potential of MNM-DE and MNM-DE-CB. Error bars indicate standard deviation from the mean based on five independent replicates.

Next, the optimal synthetic ratio of Fe^3+^ to DE was investigated to achieve good morphology, high magnetization, and an improved surface area for MNM-DE. A MNM-DE composite with a high magnetic saturation (Ms) value and surface area was obtained (Fig. 2A). Using a ratio of 0.2 (0.2 g of pure DE added to a 20 mL reaction system) Fe^3+^ to DE for fabrication, we achieved MNM-DE composites with a maximum Ms value of 42.667 emu g^−1^ and a maximum surface area of 27.67 m^2^ g^−1^ (Fig. 2A). The intensity of the magnetism of MNM-DE composites in a 0.1 ratio reaction system was higher than in a 0.3 ratio system, but overgrowth of MNM-DE caused self-aggregation, leading to mass adhesion and a decrease in the surface area of composites (Fig. 2A). FTIR was employed to confirm the structural composition of MNM-DE-CB. FTIR spectra of DE (Fig. 2B, red curve) exhibited strong peaks at 1088 cm^−1^ and 796 cm^−1^, representing stretching vibrations for Si-O-Si and vibrations for O-H^38^. After conjugation of MNM and DE, a peak appeared at 588 cm^−1^ (Fig. 2B, green curve), corresponding to Fe-O vibrations of MNM^39^. In spectra of pure CB (Fig. 2B, black curve), peaks at 2998 cm^−1^ and 2922 cm^−1^ corresponded to stretching vibrations of C-H bonds for methylene. The peak at 1470 cm^−1^ corresponded to methylene bending vibrations of C-H bonds. Finally, after conjugation of MNM-DE and CB, the allophanyl C=O peak at 1739 cm^−1^ for pure CB was shifted to 1730 cm^−1^, which implied the existence of hydrogen bonding between CB and MNM-DE^40^. Taken together, these results confirmed that the MNM-DE surface was successfully coated by CB. Next, the surface area and pore volume of MNM-DE-CB composites were characterized by N_2_ sorption measurements at 77.3 K (Fig. 2C). The surface area of MNM-DE-CB was calculated to be 46.34 m^2^ g^−1^, much larger than those of DE (2.05 m^2^ g^−1^) and MNM-DE (11.46 m^2^ g^−1^). On the other hand, the pore volume of DE (0.99 cm^3^ g^−1^) was much larger than those of MNM-DE (0.06 cm^3^ g^−1^) and MNM-DE-CB (0.14 cm^3^ g^−1^). Although the surface area of MNM-DE was increased compared with DE alone, the useful surface area of MNM-DE was decreased due to facile aggregation by magnetic adsorption and surface physical adsorption. The surface area of MNM-DE-CB was therefore markedly increased by CB, which leads to improved stability and dispersibility of the 3D MNM-DE-CB complex in water. Meanwhile, nitrogen adsorption-desorption isotherm curves of DE (black line), MNM (red line), and MNM-DE-CB (blue line) confirmed differences in adsorption quantities (Fig. 2D). All samples exhibited hysteresis loops at P/P0 ranging from 0.4 to 1.0, indicating the existence of a mesoporous structure in the composites. By contrast, the blank hysteresis loop indicated few mesoporous structures in the pure DE samples^41,42^. The pore size distribution of the mesoporous samples (DE, MNM, MNM-DE-CB) calcined at 550 °C was analyzed by Barrett-Joyner-Halenda (BJH) (Fig. S2). The pore size distribution of DE was around 100 nm due to it is not mesoporous substance (Fig. S2A). On the other hand, the pore size distribution was range 20–90 nm and 45 nm for MNM and MNM-DE-CB, respectively (Fig. S2B,C). Based on the result of the pore distribution curves, the enlarged surface area of MNM-DE-CB composites was caused by the uniform and tiny pore size distribution. Isotherms curves of MNM verified the distribution of mesoporous structures by TEM (Fig. 1A). According to the typical type IV isotherm (IUPAC classification, blue)^43^, MNM-DE-CB composites are composed of mesoporous structures, resulting in the enhancement of water dispersibility and surface area.

Next, we measured the intensity of magnetism and the Zeta potential of MNM-DE-CB. The magnetic properties of MNM-DE, MNM-DE-CB, MNM-DE-CB-dye, and MNM-DE-CB (after washing with SDS) were investigated by VSM at room temperature (Fig. 2E). The results revealed superparamagnetic behavior and minimal hysteresis, remanence, and coercivity because composites consisted of ultrafine magnetite nanocrystals. Ms values were 42.667, 40.052, 36.457, and 38.039 emu g^−1^ for MNM-DE, MNM-DE-CB, MNM-DE-CB-dye, and MNM-DE-CB (SDS wash), respectively. The Ms value for MNM-DE-CB (green curve) was slightly lower than that of MNM-DE (black curve), which might be due to the density of MNM in composites after modification with CB. It is worth noting that the modified composites still displayed strong magnetization (Fig. 2E, inset), indicating high water dispersibility and stability for MNM-DE-CB during magnetic separation and targeting. In addition, the Zeta potentials of MNM-DE and MNM-DE-CB were measured by DLS (Fig. 2F). The MNM-DE surface was coated by negatively charged ions, but MNM-DE-CB possesses positively charged ions on the surface. Based on these properties, the mesoporous structure of MNM-DE-CB may increase the physical adsorption of dye molecules. The positive charge distribution on MNM-DE-CB increased the uptake of dye molecules via electrostatic adsorption. In addition, the magnetic properties of the mesoporous magnetic nanomulberry-shaped Fe_3_O_4_ make the MNM-DE-CB composites more useful in solution. Taken together, the results confirmed the advantages of MNM-DE-CB composites compared with other composites such as MNM-DE.

### Efficient dye removal by MNM-DE-CB

**Figure 3:**
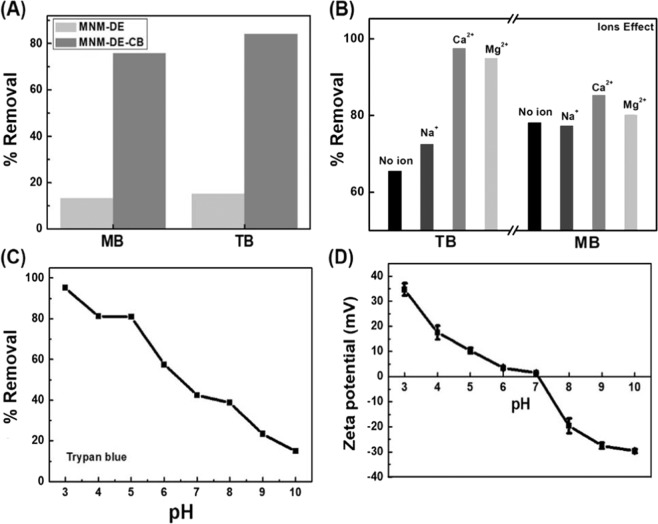
Verification of MNM-DE-CB. (**A**) Comparison of MNM-DE and MNM-DE-CB for dye removal: C0(MB) = 75 mg/L, C0(TB) = 20 mg/L, absorbent mass = 2 mg, pH = 7, volume = 1 mL, temperature = 25 °C, contact time = 1 h. (**B**) Effect of metal cations on dye removal: C0 (MB) = 75 mg/L, absorbent mass = 2 mg, pH = 7, volume = 1 mL, temperature = 25 °C; C0 (TB) = 34 mg/L, absorbent mass = 6 mg, pH = 7, volume = 1 mL, temperature = 25 °C, 0.01 mL 0.01 M NaCl, CaCl_2_, MgCl_2_. (**C**) Effect of pH on TB removal: C0 (TB) = 73 mg/L, absorbent mass = 8 mg, volume = 1 mL, temperature = 25 °C, contact time = 1 h. (**D**) Zeta potential of MNM-DE-CB at different pH values. Error bars indicate standard deviation from the mean based on five independent replicates.

To test the usefulness of MNM-DE-CB in aqueous solution, the dye removal efficiency of MNM-DE and MNM-DE-CB composites was investigated. For multiple dye removal experiments, dye solutions were measured using UV-visible spectroscopy after treating with MNM-DE or MNM-DE-CB for 1 h and rapid collection of composites by a magnet (Fig. 3A). The adsorption efficiencies toward MB (C_0_(MB) = 75 mg/L) and TB (C_0_(TB) = 20 mg/L) for MNM-DE-CB composites were 6−7 times higher than those of MNM-DE. This indicates two interaction mechanisms between dyes and MNM-DE-CB, such as host–guest interactions and physical adsorption. The MNM-DE surface was found to be negatively charged (Fig. 2F), which indicates that negatively charged MB and TB could not be adsorbed due to electrostatic repulsion. Therefore, the slight decrease in dye solution absorption intensity may be attributed to physical adsorption by the mesoporous structure of MNM-DE. On the other hand, the dye removal efficiency of MNM-DE-CB was higher than that of MNM-DE. Thus, the dye removal efficiency of positively charged MNM-DE-CB for negatively charged MB and TB could be enhanced due to electrostatic interactions.

Additionally, the distinct cavity structure may possess the ability to capture dye molecules in the presence of CB. To explore optimal conditions for MNM-DE-CB, the effects of metal cations on dye removal were examined (Fig. 3B). Due to the range of effects of metal cations and pH on Trypan Blue (TB), we used the certain amounts of composites, and increased the initial concentration of TB. Various types of metal ions such as 0.01 M NaCl, CaCl_2_, and MgCl_2_ were added to 1 mL MB (75 mg/mL) and TB (34 mg/mL) solutions containing MNM-DE-CB composites. Consistent to the previous report that dye sorption onto cucurbituril is strongly enhanced by Ca2 + for all studied dyes^44^, all metal cations enhanced the dye removal efficiency of MNM-DE-CB composites and sorption of Ca^2+^ cations proved most effective for removal of both CB and TB. The facilitate dye adsorption onto MNM-DE-CB composites might be due to the reduction of electrostatic repulsion. Despite we could not determine the specific reason regarding the differences between TB and MB, the removal efficiency of TB was improved (from 65.23% to 97%) much more than that of MB (from 78.86% to 85.39%; Fig. 3B). Although molecular weight of TB (872.88 g/mol) was bigger than that of MB (799.814 g/mol), the electron distribution of both dye molecules on the cucurbituril was similar^45,46^. Therefore, further study would be desired for investigation of the reason the differences of the removal efficiency between both dyes.

Furthermore, pH also plays a role in dye removal in aqueous solution. Since the color of MB changes from blue to red and eventually transparent between pH 9 and 14, we tested the removal rate of TB at different pH values. The adsorption efficiency of MNM-DE-CB gradually increased with decreasing pH from 10 to 2 (Fig. 3C). This could be due to a switch from a positive to a negative charged surface of MNM-DE-CB from acidic to alkaline conditions, consistent with the Zeta potential values at different pH values (Fig. 3D). In acidic and alkaline conditions, electrostatic interactions were more obvious due to significant changes in surface charge, resulting in an increase in adsorption capacity under acidic conditions, and a loss under alkali conditions.

**Figure 4:**
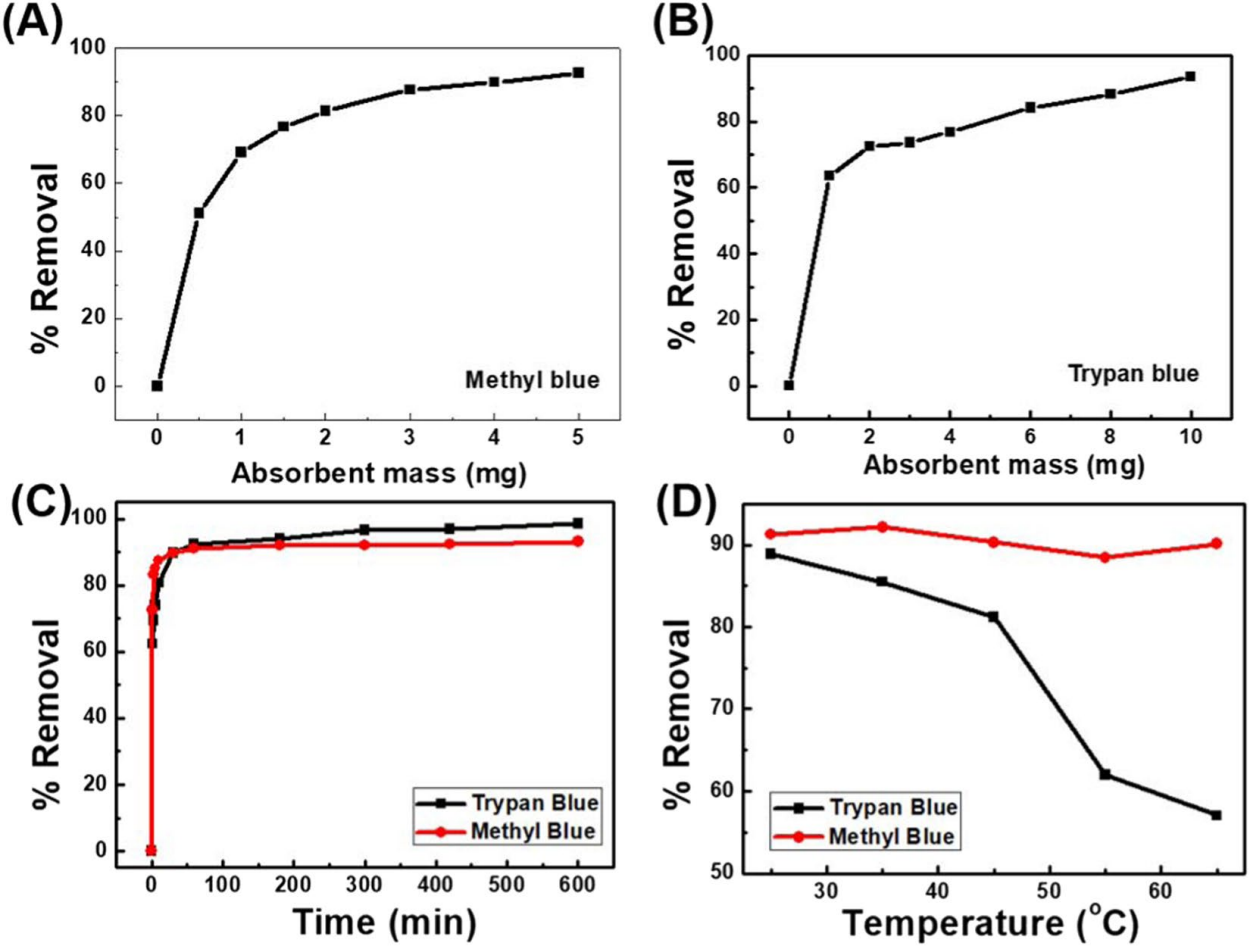
Application of MNM-DE-CB. Effects of dosage of MNM-DE-CB composites on dye removal for (**A**) Methyl Blue (MB) and (**B**) Trypan Blue (TB): C0(MB) = 75 mg/L, C0(TB) = 20 mg/L, pH = 7, volume = 1 mL, temperature = 25 °C, contact time = 1 h. (**C**) Kinetics of MB/TB removal by MNM-DE-CB: C0(MB) = 75 mg/L, absorbent mass = 3 mg, pH = 7, volume = 1 mL, temperature = 25 °C; C0(TB) = 67 mg/L, absorbent mass = 8 mg, pH = 7, volume = 1 mL, temperature = 25 °C. (**D**) Effects of temperature on dye removal: C0(MB) = 75 mg/L, MNM-DE-CB mass = 4 mg, pH = 7, volume = 1 mL, contact time = 1 h; C0 (TB) = 34 mg/L, absorbent mass = 8 mg, pH = 7, volume = 1 mL, contact time = 1 h.

Next, we evaluated the effect of adsorbent dosage on dye removal. The removal rates for MB and TB were increased gradually with an increasing amount of adsorbent (Fig. 4A,B). The maximum removal rates for MB (75 mg/L, 92.60%) and TB (20 mg/mL, 97.10%) were achieved in the presence of 5 mg and 10 mg MNM-DE-CB. The adsorption time for each dye was also evaluated (Fig. 4C). The adsorption of both MB and TB was >90% within 1 h, and saturated within 6 h. The adsorption capacity (*q*_*e*_) of MB and TB was calculated to be 186.24 mg/g and 7.88 mg/g, respectively, using Eq. (2). The removal efficiency for TB was dramatically decreased with increasing temperature, but there was only a slight decrease for MB. These results are consistent with those of a previous report^47^. Adsorption of CB was not properly conducted at higher temperature because host–guest interactions are strongly exothermic (Fig. 4D).

### Reproducibility and large volume testing of MNM-DE-CB

**Figure 5:**
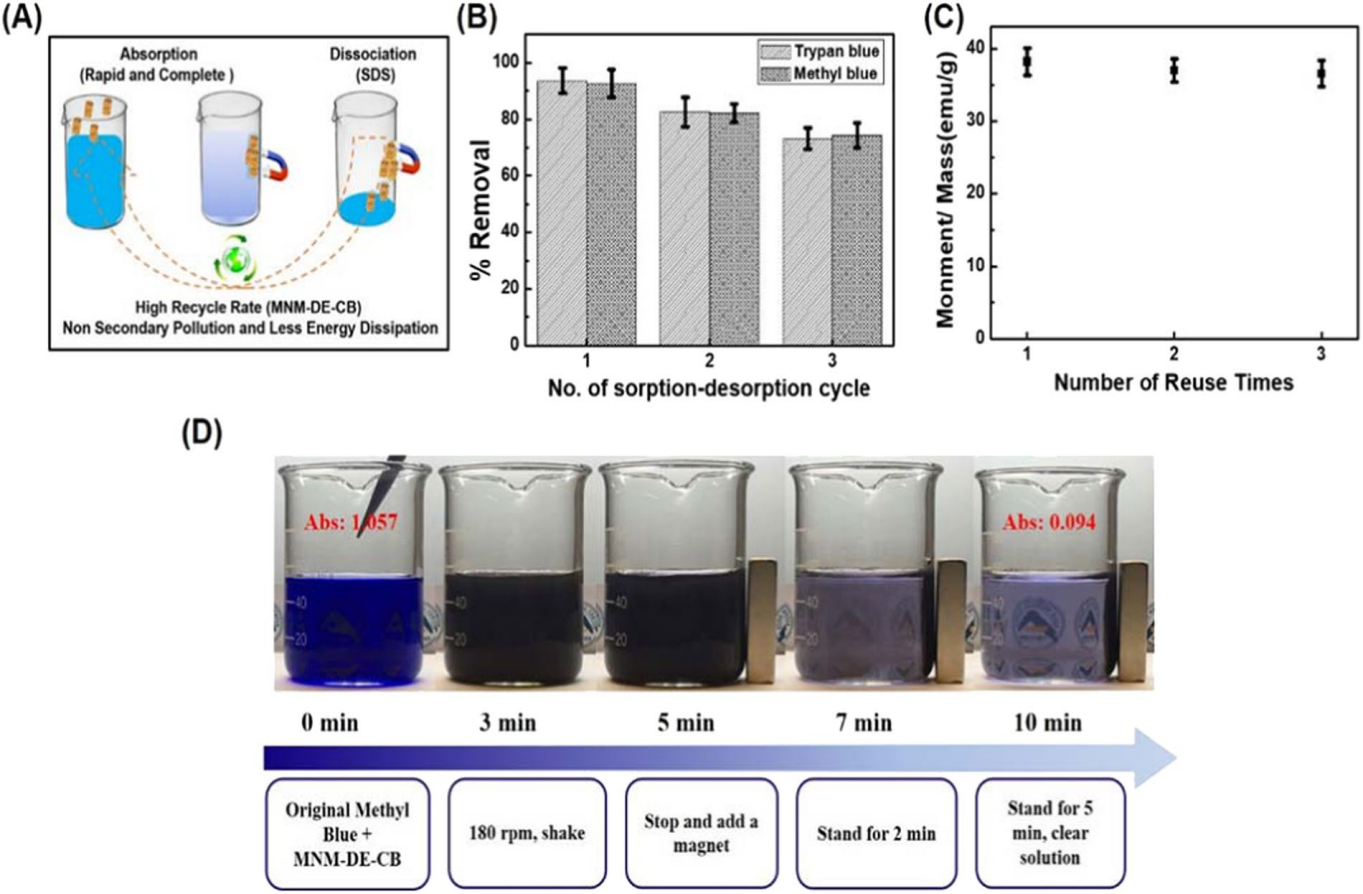
Reproducibility and 50 mL volume testing of MNM-DE-CB composites. (**A**) Diagram of dye removal and recycling by MNM-DE-CB. (**B**) Cyclic regeneration of MNM-DE-CB composites for TB and MB removal with 10% SDS as the desorption solvent. (**C**) Ms value of MNM-DE-CB following reuse. (**D**) Dye removal by MNM-DE-CB: MB = 75 mg/L, absorbent mass = 30 mg, volume = 50 mL, temperature = 25 °C.

Due to the magnetic properties of the adsorbent, pollutants can be rapidly collected by a magnet, and adsorbed dyes and other molecules can be dissociated from the adsorbent using SDS. Figure 5A shows the process of absorption, dissociation, and efficient recycling with minimal secondary pollution and low energy loss. The reusability of MNM-DE-CB was evaluated using 10% SDS as the desorbing agent (Fig. 5B). The dye removal efficiency was slightly decreased from 93.66% to 73.25% for TB, and from 92.60% to 74.25% for MB, after three uses due to incomplete dye removal from the surface of MNM-DE-CB.

Meanwhile, the magnetic properties of the adsorbent did not change significantly after three times uses based on the Ms values (Fig. 5C). To test the long-term stability and recyclability of MNM-DE-CB by magnetization, we used sodium dodecyl sulfate (SDS) to remove bound dye molecules and performed rebinding experiments. Remarkably, magnetization of MNM-DE-CB (2 mg) continued to work after being recycled 60 times through repeated washing and reabsorption of dye molecules for 1 month (Fig. S3). However, Ms values of MNM-DE-CB decreased continuously after recycling, possibly because absorbed dye molecules were not completely desorbed from the adsorbent by SDS. Despite this shortcoming, the recyclability of MNM-DE-CB for dye removal represents a significant advantage for large scale industrial applications.

In addition, we performed MB removal testing at a larger volume scale (50 mL and 1 L) to confirm the potential usefulness in various industries (Fig. 5D and Fig. S4). A 30 mg sample of MNM-DE-CB was used for MB dye (75 mg/L) removal. After reaction of MB and the adsorbent for 5 min, the adsorbent was collected using a magnet for 5 min. Within this time, the MB dye solution was almost 96% cleared (Abs0 from 1.057 to 0.094) at the 50 mL scale, indicating that the adsorbent achieved high water dispersibility and dye removal efficiency in the increased volume. Furthermore, the MB dye solution was ~75% cleared (Abs0 from 0.463 to 0.117) after 10 min at the 1 L volume scale (Fig. S4). These results demonstrate the acceptable reproducibility of MNM-DE-CB, as well as rapidity, simplicity, and low cost at a larger scale.

### Biocompatibility and drug testing of MNM-DE-CB

**Figure 6:**
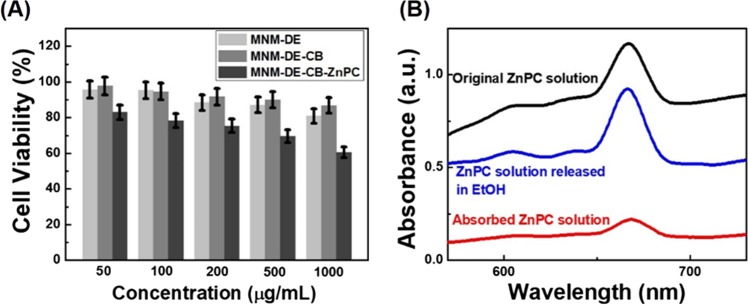
Biocompatibility testing of MNM-DE-CB. (**A**) Cytotoxicity testing based on cell viability of HCT-116 cells after incubating with different concentrations of MNM-DE or MNM-DE-CB for 24 h at 37 °C. (**B**) ZnPC drug loading-release testing. UV/visible absorption spectra of original, absorbed, and final ZnPC solutions after being released with ethanol for 24 h.

The biocompatibility of nanomaterials is an essential property for biomedical applications, especially inherent toxicological issues^48^. In the present study, the cytotoxicity of MNM-DE and the MNM-DE-CB was evaluated using MTT assays in colorectal adenocarcinoma HCT-116 cells. A series of concentrations of MNM-DE and MNM-DE-CB ranging from 50 to 1000 μg/mL were incubated with HCT-116 cells for 24 h. Cell viability was not adversely affected (83−97%) at concentrations up to 1 mg/mL for both composites (Fig. 6A).

Meanwhile, to demonstrate the drug delivery applicability of MNM-DE-CB, we employed this adsorbent as a drug carrier with zinc phthalocyanine (ZnPC) as a drug delivery model. The absorption and release efficiencies for ZnPC with MNM-DE-CB were 81.89% and 96.84%, respectively (Fig. 6B). When MNM-DE-CB-ZnPC was added to HCT116 cells, the viability of cancer cells was significantly decreased compared with MNM-DE-CB alone. Thus, MNM-DE-CB is a biocompatible and non-toxic material that could prove useful as a drug delivery system in clinical applications.

## Methods

### Chemicals and reagents

Calcined DE, iron chloride hexahydrate (FeCl_3_.6H_2_O), sodium acetate (NaAc), ethylenediamine (EDA), ethylene glycol (EG), cucurbit[6]uril hydrate, sodium dodecyl sulfate (SDS), sodium chloride (NaCl), calcium chloride (CaCl_2_), magnesium chloride (MgCl_2_), Methyl Blue, Rhodamine B, Methylene Blue, Thiazolyl blue tetrazolium bromide (MTT), and zinc phthalocyanine (ZnPC) were purchased from Sigma Aldrich (Saint Louis, Missouri, USA). Trypan Blue stain (0.4%), Dulbecco’s modified Eagle’s medium (DMEM, 1×), fetal bovine serum (FBS), and phosphate-buffered saline (PBS) were obtained from Gibco (Big Cabin, Oklahoma, USA). Dimethyl sulfoxide (DMSO) was acquired from EMD Millipore Corp (Darmstadt, Germany). The human colorectal carcinoma cell line HCT-116 (ATCC®CCL-247) was purchased from ATCC (Manassas, VA, USA). All materials were used as received with no further treatment.

### Preparation of magnetic nanomulberry-diatomaceous earth (MNM-DE)

Magnetic nanomulberry-diatomaceous earth (MNM-DE) was synthesized using a typical hydrothermal method^35^. Firstly, DE was purified by gravity settling in deionized (DI) water, and 1.0 g FeCl_3_.6H_2_O and different amounts of pure DE (0.3 g, 0.2 g, or 0.1 g) were added to 20 mL EG and stirred for 30 min to generate three brownish-yellow solutions. Following addition of 3.0 g C_2_H_3_NaO_2_ and 10 mL EDA, the mixture was stirred for 30 min with shaking at 1000 rpm, and then sealed in a 100 mL Teflon-lined stainless-steel autoclave at 200 °C for 8 h and allowed to cool to room temperature. The resulting black products were washed several times with water, collected by a magnet, and dried at 50 °C in a vacuum oven. Finally, 0.7 g MNM-DE was obtained from each reaction (~60–75% of coating ratio).

### Preparation of magnetic nanomulberry-diatomaceous earth-cucurbit[6]uril (MNM-DE-CB)

Preparation of MNM-DE-CB was performed by the microwave method. Briefly, 5 mg MNM-DE was dissolved in 1 mL DI water to form a 5 mg/mL MNM-DE solution. A 25 mg sample of CB[6] was then added to 500 μL DI water and sonicated for 1 min using an ultrasonic instrument. Subsequently, 25 μL of the 50 mg/mL CB solution was added dropwise to 2 mL MNM-DE solution and heated in a microwave oven for 1 min.

### Characterization

The morphology of MNM, MNM-DE, and MNM-DE-CB was characterized using field-emission scanning electron microscopy (FE-SEM) on a JSM-7500F instrument (JEOL) and transmission electron microscopy (TEM) on a G2 F30 instrument (Tecnai) to confirm the MNM structure and decoration of MNM on DE. Brunauer-Emmett-Teller (BET) surface areas and nitrogen adsorption-desorption isotherms of MNM, MNM-DE, and MNM-DE-CB were determined by nitrogen sorption-desorption on an ASAP 2020 V3.04 H accelerated surface area and porosimetry system (Micromeritic) at 77.3 K. Zeta potentials of materials were measured using dynamic light scattering (DLS) on a DynaPro NanoStar instrument (Wyatt). Fourier-transform infrared spectroscopy (FTIR) analysis was performed using a JASCO 6300 instrument (JASCO) on bare MNM, MNM-DE, and MNM-DE-CB to obtain information on chemical modification. Hysteresis loops were collected on a vibrating sample magnetometer (VSM) using a LakeShore 7404 instrument (LakeShore) at room temperature.

### Adsorption equilibrium experiment

For dye adsorption, Methyl Blue (MB) and Trypan Blue (TB) were tested. Different amounts of MNM-DE-CB were added to 1 mL dye solution at a defined concentration in a 1.5 mL tube. The mixture was shaken continuously for 1 h at room temperature, and adsorbents were collected with a magnet. The concentration of residual dye remaining in solution was analyzed by UV-Vis spectroscopy on a UV-2550 instrument (Shimadzu) by measuring the absorbance at the wavelength of maximum absorption. The % removed (*R*) and adsorption capacity (*q*_*e*_) were calculated using the following equations:

1 $$R=\frac{({C}_{o}-{C}_{e})}{{C}_{o}}\times 100 \% $$

2 $${q}_{e}=\frac{({C}_{o}-{C}_{e})\,V}{m}$$

where *C*_*o*_ and *C*_*e*_ are the initial and equilibrium concentrations of dyes (mg/L), *q*_*e*_ is the equilibrium adsorption capacity (mg/g), m is the mass of MNM-DE or MNM-DE-CB (g), and *V* is the volume of solution (L). For regeneration measurements, recycled adsorbents were washed with 10% SDS, collected using a magnet, and used for subsequent adsorption experiments.

### *In vitro* cytotoxicity assay

For cell culture, colorectal adenocarcinoma cell line HCT-116 cells were cultured in DMEM medium Supplemented with 10% FBS at 37 °C in an atmosphere with 5% CO_2_ and 95% relative humidity. In each well of a 96-well plate 100 μL HCT-116 cells were seeded at a density of 5×10^4^ cells/well and allowed to attach for 24 h. The medium has been removed and the cells were washed once with PBS. MNM-DE-CB, MNM-DE, and DE at concentrations of 50, 100, 200, 500, or 1000 μg/mL in DMEM were added to separate wells in octuplicate and incubated with cells for 6 or 24 h. Corresponding samples of MNM at concentrations of 4, 8, 16, 40, and 80 μg/mL in DMEM were subjected to the same process. After incubation, suspensions have been removed and wells were washed once with PBS. A 100 μL sample of MTT (0.5 mg/mL in culture medium) was then added, and cells were incubated for 4 h at 37 °C in 5% CO_2_ prior to analysis. The medium was removed and 150 μL DMSO was added to dissolve blue formazan crystals. The absorbance of the resulting dye was measured at 490 nm using a microplate reader (BioTek, US). Absorbance values for untreated cells were taken as controls (100% survival). Cell viability was then calculated according to the following equation:

3 $$({\rm{absorbance}}\,{\rm{of}}\,{\rm{test}}\,{\rm{cells}})/({\rm{absorbance}}\,{\rm{of}}\,{\rm{control}}\,{\rm{cells}})\times 100 \% $$

For drug testing, MNM-DE-CB (40 mg) was added to 5 mL ZnPC/EtOH solution (2 mM) at room temperature. The mixture was shaken for 24 h, and drug-loaded composites were separated and tested by MTT assay.

## Conclusions

We herein demonstrate the synthesis of an adsorbent composed of magnetic functionalized DE and CB composites possessing rapid absorption ability and high solubility in aqueous solution. The MNM-DE-CB composite was synthesized by a two-step method that involved growing magnetic rods on the surface of DE, followed by CB coating through hydrogen bonds, which improved the solubility of MNM-DE in aqueous solution. The MNM-DE-CB composite is a highly water-dispersible and efficient adsorbent for the removal of dyes (MB and TB), possesses an increased surface area, and operates via three effective mechanisms: physical adsorption, electrostatic interactions, and host–guest interactions.

Various potentially influential factors were investigated, and MNM-DE-CB displayed high adsorption capacity in acidic solution and in the presence of metal cations at room temperature. Using this novel adsorbent has a rapid turnaround time due to the ability to recover using a magnet, making it suitable for large sample volumes. Thus, it is a promising alternative for the removal of toxic pollutants in several industries including environmental remediation and drinking water preparation. In addition, *in vitro* cytotoxicity assays were performed to confirm that MNM-DE-CB composites behaved in a dose-dependent manner, and no significant cytotoxicity was observed, making them good candidates for drug delivery. The synthesis of MNM-DE-CB in the present work was at the milligram scale, and further study at the gram scale is needed to prepare for various applications. We believe that this strategy for preparing MNM-DE-CB will open a new avenue for reducing magnetic self-precipitation and facilitate the efficient utilization of super-molecular CB in aqueous applications at large scale. Specifically, the biocompatibility and effective molecular encapsulation ability of MNM-DE-CB may lead to uses in drug delivery. Furthermore, the MNM-DE-CB with the absorption property using magnetic field could be applied to clinical applications such as biomolecules (protein, nucleic acids) isolations for disease diagnostics (protein and nucleic acids isolation) and treatments (bone space therapy, and local cancer therapy).

## Supplementary information

supplementary file.
