# Supplementary material for: An enhanced recyclable 3D adsorbent for diverse bio-applications using biocompatible magnetic nanomulberry and cucurbituril composites
Source: Sci Rep. 2020 Jan 16;10:443. doi: 10.1038/s41598-019-57336-4 (PMC6965109; doi:10.1038/s41598-019-57336-4)
Supplement: Supplementary file 1 — supplementary file. [file 41598_2019_57336_MOESM1_ESM.docx]

**Supplementary**

**An enhanced recyclable 3D adsorbent for diverse bio-applications using biocompatible magnetic nanomulberry and cucurbituril composites**

Yange Luan^1,+^, Huifang Liu^1,+^, Zhen Qiao^1^, Bonhan Koo^1^, Jaehyub Shin^1^, Yoon Ok Jang^1^, Jin-Seo Noh^2^, Yong Shin^1,*^

^1^Department of Convergence Medicine, Asan Medical Institute of Convergence Science and Technology (AMIST), University of Ulsan College of Medicine, Biomedical Engineering Research Center, Asan Institute of Life Sciences, Asan Medical Center, 88 Olympicro-43gil, Songpa-gu, Seoul, Republic of Korea.

^2^Department of Nano-Physics, Gachon University, 1342 Seongnamdaero, Sujeong-gu, Gyeonggi-do 13120, Republic of Korea

^+^ These authors (Y.L. & H.L.) contributed equally to this work.

^*^To whom correspondence should be addressed. Tel: +82-2-3010-4193; E-mail: [shinyongno1@gmail.com](mailto:shinyongno1@gmail.com)


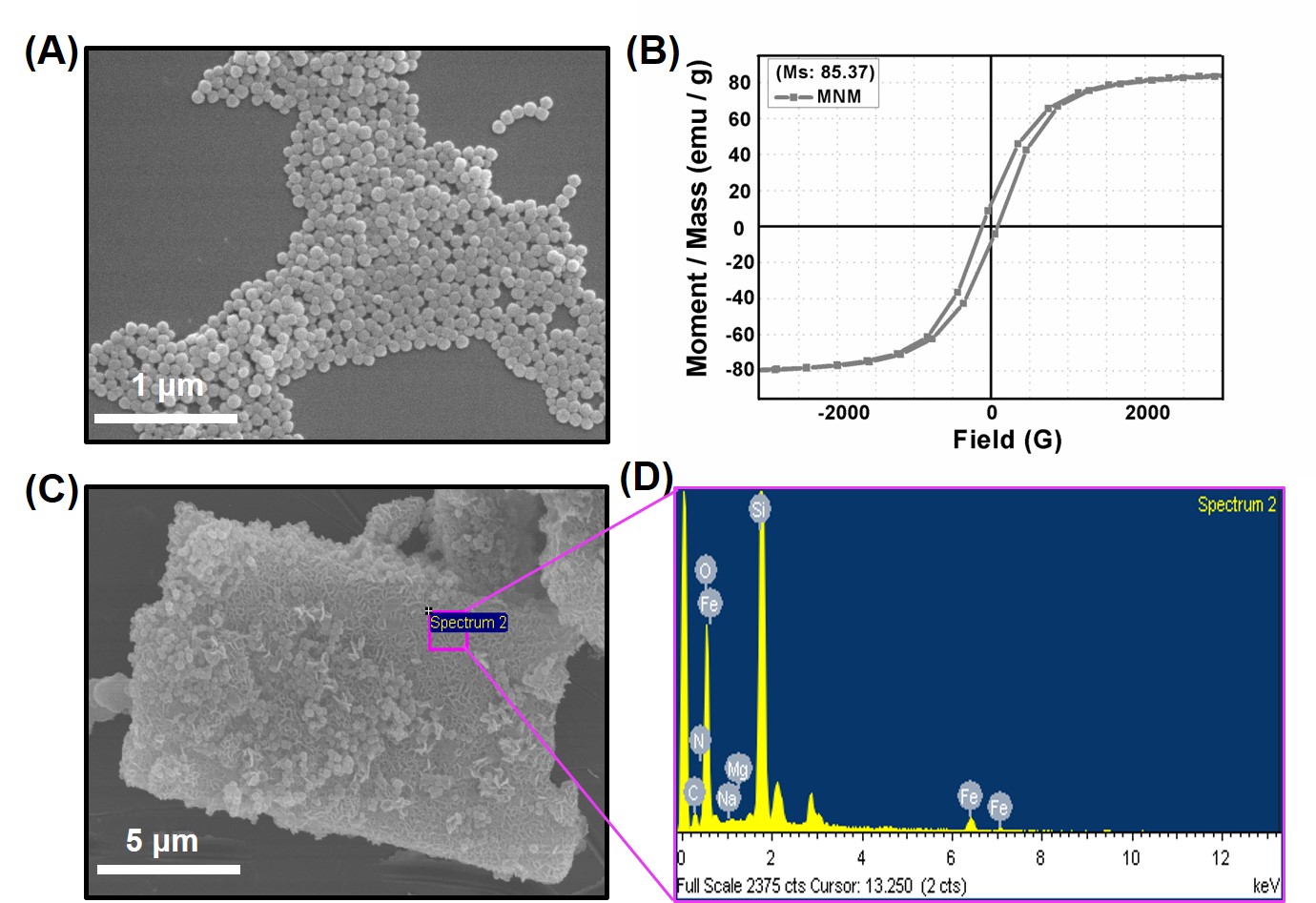


**Figure S1.** **Size characterization of MNM-DE-CB.** (A) SEM image of MNM. (B) Magnetic hysteresis loops of MNM. (C) SEM image of MNM-DE (D). Energy-dispersive X-ray spectroscopy (EDX) of MNM-DE.


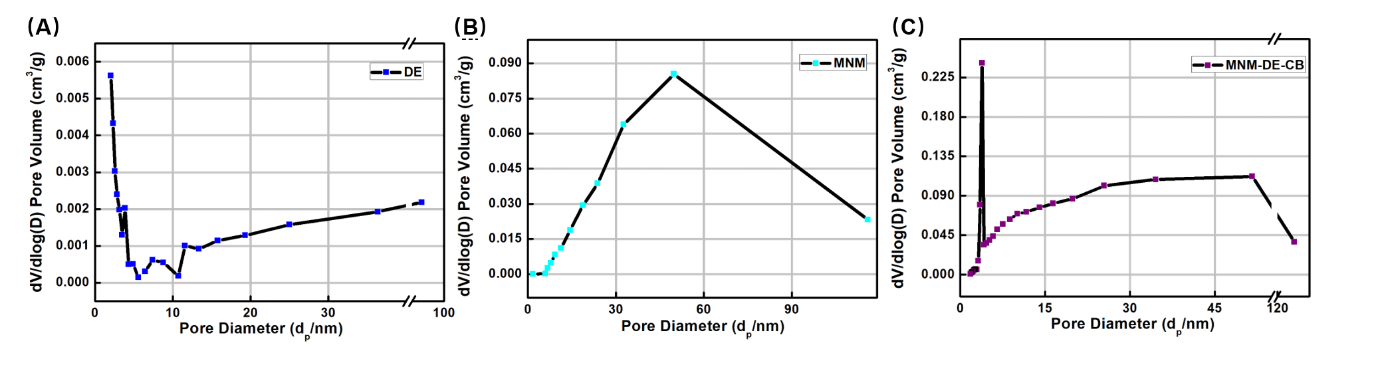
**Figure S2. Pore-size distribution curve of** **DE (A), MNM (B), MNM-DE-CB (C) samples.**

**Figure S3. Magnetic saturation (Ms) of MNM-DE-CB for long-term stability and recyclability.** Conditions included an absorbent mass of 2 mg, pH 7, a volume of 1 mL, and 25°C.


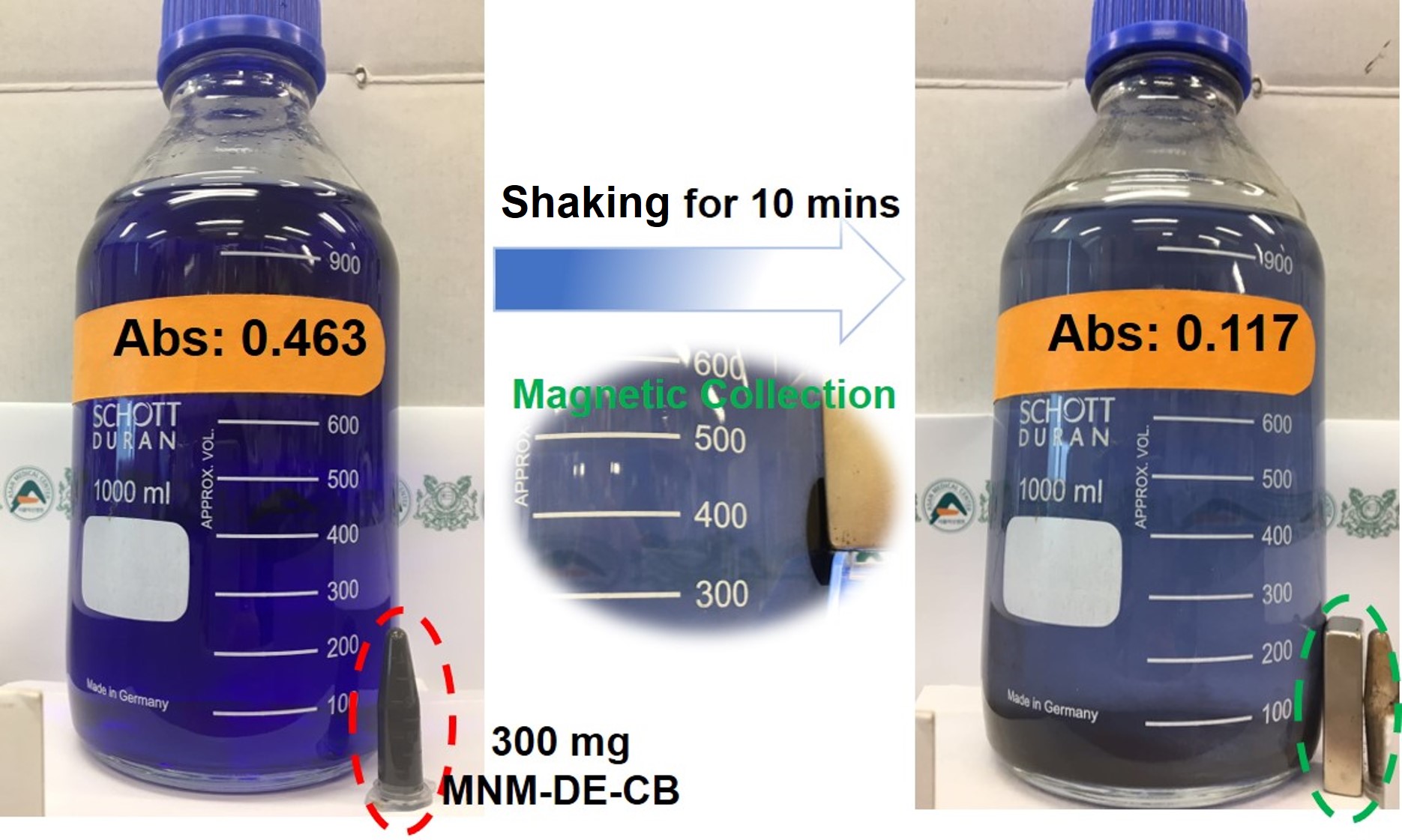


**Figure S4.** **Analysis of MNM-DE-CB composites at a larger volume (1 L) scale.** Dye removal by MNM-DE-CB (MB = 37.5 mg/L, absorbent mass = 300 mg, volume = 1 L, temperature = 25°C).
